# Genomic and Phenotypic Biomarkers for Precision Medicine Guidance in Advanced Prostate Cancer

**DOI:** 10.1007/s11864-023-01121-z

**Published:** 2023-08-10

**Authors:** Fatemeh Davoudi, Afshin Moradi, Therese M. Becker, John G. Lock, Brian Abbey, Davide Fontanarosa, Annette Haworth, Judith Clements, Rupert C. Ecker, Jyotsna Batra

**Affiliations:** 1 School of Biomedical Sciences, Faculty of Health, Queensland University of Technology, Brisbane, 4059 Australia; 2 Department of Medical Genetics, School of Medicine, Ahvaz Jundishapur University of Medical Sciences, Ahvaz, Iran; 3 Centre for Genomics and Personalised Health, Queensland University of Technology, Brisbane, 4059 Australia; 4 Translational Research Institute, Queensland University of Technology, Brisbane, 4102 Australia; 5 Ingham Institute for Applied Medical Research, University of Western Sydney and University of New South Wales, Liverpool, 2170 Australia; 6 School of Biomedical Sciences, Faculty of Medicine and Health, University of New South Wales, Sydney, 2052 Australia; 7 Department of Mathematical and Physical Sciences, School of Computing Engineering and Mathematical Sciences, La Trobe Institute for Molecular Sciences, La Trobe University, Bundoora, VIC Australia; 8 School of Clinical Sciences, Queensland University of Technology, Gardens Point Campus, 2 George St, Brisbane, QLD 4000 Australia; 9 Centre for Biomedical Technologies (CBT), Queensland University of Technology, Brisbane, QLD 4000 Australia; 10 Institute of Medical Physics, School of Physics, University of Sydney, Camperdown, NSW 2006 Australia; 11 TissueGnostics GmbH, EU 1020 Vienna, Austria

**Keywords:** Advanced prostate cancer, Genotypic biomarkers, Phenotypic biomarkers, Precision medicine

## Abstract

Prostate cancer (PCa) is the second most diagnosed malignant neoplasm and is one of the leading causes of cancer-related death in men worldwide. Despite significant advances in screening and treatment of PCa, given the heterogeneity of this disease, optimal personalized therapeutic strategies remain limited. However, emerging predictive and prognostic biomarkers based on individual patient profiles in combination with computer-assisted diagnostics have the potential to guide precision medicine, where patients may benefit from therapeutic approaches optimally suited to their disease. Also, the integration of genotypic and phenotypic diagnostic methods is supporting better informed treatment decisions. Focusing on advanced PCa, this review discusses polygenic risk scores for screening of PCa and common genomic aberrations in androgen receptor (AR), PTEN-PI3K-AKT, and DNA damage response (DDR) pathways, considering clinical implications for diagnosis, prognosis, and treatment prediction. Furthermore, we evaluate liquid biopsy, protein biomarkers such as serum testosterone levels, SLFN11 expression, total alkaline phosphatase (tALP), neutrophil-to-lymphocyte ratio (NLR), tissue biopsy, and advanced imaging tools, summarizing current phenotypic biomarkers and envisaging more effective utilization of diagnostic and prognostic biomarkers in advanced PCa. We conclude that prognostic and treatment predictive biomarker discovery can improve the management of patients, especially in metastatic stages of advanced PCa. This will result in decreased mortality and enhanced quality of life and help design a personalized treatment regimen.

## Introduction

Prostate cancer (PCa) is a complex health issue. According to the World Health Organization (WHO) in 2020, PCa is the third most frequent malignancy and the second most common neoplasm diagnosed in men worldwide. With yearly 1,414,259 new cases, PCa is the third leading cause of cancer death with 375,304 new deaths worldwide [1]. It is the fifth ranked cause of cancer associated death in males, and once advanced to metastatic disease, patients have a poor prognosis with a 5 year survival of ~ 30% [1, 2]. Indeed, the majority of patients eventually acquire resistance to first- and second-line androgen-deprivation therapies (ADT), progressing to castration-resistant prostate cancer (CRPC) within approximately 5 years from diagnosis [3].

Prostate-specific antigen (PSA) is currently the main diagnostic and prognostic biomarker for PCa. Screening and monitoring the disease using PSA to guide patient management have significantly reduced PCa mortality due to earlier detection and the ability to predict progression (Table 1) [4]. PSA expression is regulated by androgen receptor (AR), and it is normally synthesized in epithelial cells of the prostate gland with abnormally high levels implicated in tumor recurrence in men with PCa [36, 37]. Accordingly, PCa diagnosis has dramatically increased over time following the introduction of PSA screening; however, the PSA test alone is suboptimal and agreement on accuracy remains elusive. PSA lacks adequate sensitivity and specificity in distinguishing PCa and non-malignant prostate lesions, such as benign prostatic hyperplasia (BPH) and prostatitis, requiring other supportive diagnostic approaches in the clinic [38]. A range of additional commercial biomarkers has emerged, including the Prostate Health Index (PHI), the 4-kallikrein score (4Kscore), SelectMDx, ExoDx Prostate Intelliscore (EPI), and MyProstateScore (MPS) as well as Decipher™. These biomarkers are intended to improve the performance of serum PSA, providing more accurate assessments of disease aggressiveness, minimizing recourse to invasive biopsies, and facilitating more optimal therapeutic disease management overall (Table 1) [5, 7]. Metastatic prostate cancer (mPCa) comprises a spectrum of diverse genotypes and phenotypes. Notwithstanding advances in screening and treatment of PCa, active investigation continues to seek improved prognostic and treatment predictive biomarkers, as well as new effective therapeutic targets. Precision medicine in advanced PCa aims to diversify and more optimally target treatment strategies as well as accelerate metastatic cancer detection at earlier stages. Thus, currently emerging phenotypic and genomic biomarkers, combined with established and emerging medical imaging technologies, aim to enable improved clinical risk stratification and precision medicine in this disease. Hereafter we summarize recent advancements in advanced PCa biomarker discovery, considering two major categories—genomic and phenotypic biomarkers.

**Table 1:** Predictive/prognostic biomarkers and screening tests in management of advanced PCa

Class	Marker	Description	Clinical utility	Ref
Mainstream diagnostic markers Clinically and commercially available	PSA	First-Line treatment	Screening and monitoring PCa	[4]
	Total PSA, free-PSA, p2PSA isoform/serum	Prostate Health Index (PHI)	Prediction of high grade PCa Avoiding unnecessary biopsies Improve the performance of serum PSA	[5, 6]
	Total PSA, free-PSA, intact PSA, hK2 /serum	4-kallikrein score (4Kscore)	Risk prediction for mPCa Avoiding unnecessary biopsies	[5, 6]
	HOXC6 and DLX1 mRNA/post-DRE urine	SelectMDx	Predictive for high grade PCa	[5, 6]
	Exosomal level of RNA expression of three genes (e.g., SPDEF, ERG and PCA3)/urine	ExoDx Prostate Intelliscore (EPI)	predictive for the probability of high-grade PCa from Grade Group (GG) 2 or higher	[5, 6]
	PCA3 and TMPRSS2-ERG mRNA, PSA/post-DRE urine	MyProstateScore (MPS)	Avoiding unnecessary biopsies	[5, 6]
	22-gene microarray-based genomic classifier (GC)	Decipher (post-PR testing)	Risk estimating for metastases development	[7]
Genotypic biomarkers	Genome-wide association studies (GWAS)	High-throughput genotyping	Uncovering the genomic variants associated-risk in developing a trait/disease	[8]
	Polygenic risk scores (PRSs)	Numerical indicators	Estimating the individual's genetic liability to a trait/disease	[9]
	AR point mutations, AR amplifications, and AR variants HSD3B1(1245C) genotype	Androgen receptor (AR)	Potentially predictive for optimal treatment Worse OS and early development of CRPC Potentially predictive for time of progression to CRPC in response to ADT	[10–13] [14–17]
	PTEN loss	PTEN and PI3K-AKT pathway	Lack of response to abiraterone acetate, but a more desirable response to docetaxel Potentially predictive for AKT inhibitors Poor survival	[18, 19]
	Homologous recombination deficiency (HRD); BRCA2, ATM, CDK12, and BRCA1 variants Mismatch repair deficiency (MMRd); MSH2 and MSH6 mutations	DNA damage response (DDR)	Predictive for PARP inhibitors Worse prognosis Potentially predictive for PD-1 inhibitors Shorter OS	[20–22] [23–25]
	CTCs and ctDNA RNA-based fragments Exosomes	Reducing the necessity of invasive biopsies	Commercially available/clinical usage Clinical trial Clinical trial	[26, 27]
Phenotypic biomarkers (Clinical trials)	Serum testosterone level (pre-treatment)	Levels ≥ 5 ng/dL vs. < 5 Levels > 0.05 vs. 0.05 > ng/mL	levels ≥ 5 ng/dL; patients benefit more from AR-targeted therapy/longer PSA-PFS and OS Levels < 5; treatment with abiraterone; prolonged PSA-PFS and higher PSA response Levels > 0.05 ng/mL; treatment with enzalutamide; superior PFS, while worse PFS with docetaxel	[28] [29]
	SLFN11 expression	Overexpression of SLFN11	Predictive for platinum-based chemotherapy/longer radiographic PFS, diminishing PSA ≥ 50%	[30]
	Total alkaline phosphatase (tALP)	Normalization of tALP	prognostic for OS Treatment with Radium-223; better OS	[31]
	Neutrophil-to-lymphocyte ratio (NLR)	Higher baseline NLR NLR ≤ 5 vs. NLR > 5 NLR ≥ 2.5 vs. NLR < 2.5	Receiving abiraterone; worse OS Receiving enzalutamide; inferior OS and CSS Treatment with 223Ra; improved OS Better 1-year rPFS and 2-year CSS; received post-docetaxel ARAT agents compared to patients with pre-docetaxel ARAT Lower 1-year rPFS and 2-year CSS	[32] [33] [33]
	PD-L1 expression	Tumor pathology	Higher risk of progression Higher risk of recurrence after radical prostatectomy Immune checkpoint-inhibiting therapies	[34]
	PSMA and choline proteins	Radioligand-based imaging modalities	Detecting nodal and distant metastatic disease	[35]

## Genomic/genotypic biomarkers

Complex genetic and epigenetic factors have been implicated in the progression of PCa from indolence into aggressive disease. Yet, a comprehensive understanding of the genetic basis for this heterogeneity remains lacking. Recent developments in high-throughput genotyping and genomic sequencing have revealed recurrent alterations in mPCa-related genes, enabling more accurate classification and stratification of patients to appropriate mPCa targeted therapies [39]. Below, we describe PCa precision medicine applications of polygenic risk scores (PRS) as well as rare germline and somatic variations in critical genes (Table 1).

### Polygenic risk scores (PRSs) in PCa patient screening

Despite other risk factors implicated in the pathogenesis of PCa, advancements in high-throughput genetic analysis have illustrated a crucial role for heredity in PCa development [40]. Genome-wide association studies (GWAS) have increasingly revealed the heritability of trait-predisposing single-nucleotide polymorphisms (SNPs). A recent meta-analysis of PCa GWAS study by Conti et al. reported 86 novel genetic risk variants, bringing the total to 269 known risk variants (Table 1) [41••]. Gene-based association studies have demonstrated that utilizing GWAS to identify PCa-associated SNPs, integrated with expression quantitative trait locus (eQTL) and PRSs, provides insight into the genetic liability of traits [8]. PRSs are numerical indicators estimating the additive effects of variants spanning various risk alleles associated with a specific trait. Since PCa is a polygenic disease, PRSs have been proposed as a strong predictor for PCa risk assessment [9]. For instance, it has been shown that compared with the national average, having a PRS in the top 10% or top 1% can predict a 2.9- or 5.7-fold increase in PCa risk, respectively [42]. A multiethnic PRS study by Pylm et al. suggested that PRS analysis can be used to identify males who are at high risk for PCa, although it is not specific to the risk of aggressive disease [43]. There is no stronger association between the PRS and lethal PCa; however, it can be integrated with other specific markers for aggressive cancer types [43, 44]. Similarly, PRSs may be used to predict and stratify disease risk among men of different ethnic groups (Table 1) [10].

### Alterations in AR and its pathways

Genomic alterations in AR pathways are important PCa biomarkers with the potential to better guide current and future PCa therapies as well as improve stratification to clinical trials. AR aberrations, commonly associated with progression to CRPC, include AR gene amplifications (30–50% of CRPC), point mutations (~ 15% of CRPC) and splice variants encoding truncated proteins [11]. At progression to CRPC, PCa cells display AR overexpression and increased AR signaling [12]. Increased AR expression could result from AR gene amplifications, which are rarely observed in treatment-naive patients. This implies CRPC cell adaptation in response to ADT [13]. AR point mutations are mainly reported in the ligand-binding and N-terminal domains of AR, acquiring resistance and/or reducing affinity for anti-androgenic agents. Recurring mutations, including W742C, and T878A arise in resistance to flutamide and bicalutamide, while the F877L mutation commonly emerges after enzalutamide or apalutamide treatment [45•]. Alternate mutations may modify the affinity for endogenous or exogenous ligands with T878A mutation, creating higher affinity for progesterone and the L702H mutation for prednisone, contributing to abiraterone resistance [45•]. Notably, AR point mutations are reported for 15–20% of patients with CRPC, rising to nearly 40% of CRPC patients treated with AR antagonists [13]. AR splice variants tend to lack the last exons of AR, which encode the C-terminal ligand-binding domain. This renders AR variants (AR-Vs) constitutively active even in the absence of the ligand. Such variants are also reported to have a partially distinct target gene repertoire [46]. AR-V7 (also termed AR3), AR-V12 (also termed ARv567es), and AR-V3 are the most frequent variants (75% of all variants detected) [14]. High levels of AR–V7, AR-V12, and AR-V3 mRNA have been implicated in resistance to ADT, as well as to accelerated CRPC progression and poor overall survival (OS) [13]. Patients with AR gains-of-function are resistant to enzalutamide/abiraterone and proposed to benefit from taxane-based therapies [2]. In prostatic tissue, the 3-beta-hydroxysteroid dehydrogenase-1 (HSD3B1) enzyme, encoded by the HSD3B1 gene, catalyzes the conversion of dehydroepiandrosterone (DHEA) to testosterone and dihydrotestosterone (DHT). HSD3B1 (1245A) is an adrenal-restrictive allele, encoding a more rapidly degraded enzyme, and restricts the conversion of DHEA to DHT; in contrast, the HSD3B1 (1245C) allele is an adrenal-permissive allele encoding a more durable enzyme and leads to more DHT production. Multiple studies have demonstrated the correlation of HSD3B1 (1245C genotype) with shorter time of progression to CRPC in response to ADT in patients with metastatic hormone-sensitive PCa (mHSPC) [15–17]. A study by Hearn et al. [47] evaluated the clinical outcomes of the inheritance of HSD3B1 (1245C). The number of patients with low-volume disease freedom from CRPC at 2 years was considerably lower (51.0% vs. 70.5%), and OS at 5 years was notably worse (57.5% vs. 70.8%) compared to those with adrenal-restrictive genotype patients who had received ADT with or without docetaxel. However, no association was found with HSD3B1 genotype and high-volume disease as well as benefit from treatment with docetaxel [47].

### PTEN and PI3K-AKT pathway

PTEN/PI3K/AKT signaling constitutes a critical PCa pathway, especially in CRPC. This pathway regulates multiple cellular processes, including cell growth, proliferation, apoptosis, metabolism, and adhesion. PI3K (phosphatidylinositol-3 kinase) is a membrane-associated protein that, when activated by receptor tyrosine kinases, phosphorylates PIP2 to generate PIP3. PIP3 recruits AKT (protein kinase B) to the plasma membrane, initiating a cascade of cell responses to extracellular signals. PTEN is a tumor suppressor protein that negatively regulates PI3K/AKT signaling [48]. Loss of PTEN expression causes PIP3 accumulation and subsequent increases in AKT phosphorylation, driving persistent pathway signaling and dysregulated cellular functions [49]. Various alterations, including genomic rearrangement or mutations in AKT, PIK3CA, PIK3CB, PIK3R1, and PIK3R3, are associated with PCa development. However, loss of the PTEN locus (10q23.31) is the most common genomic aberration, occurring in nearly 40% of CRPC cases, rising to 70–80% when considering hemizygous deletions [50]. Since PTEN loss is associated with higher risk of recurrence in localized PCa after radical prostatectomy, as well as poor survival outcomes in mPCa patients, it is well-reasoned as a prognostic and predictive biomarker for responses to antitumor agents [18, 19, 51, 52]. Two retrospective studies associate PTEN loss in metastatic CRPC (mCRPC) with lack of response to abiraterone acetate, but a more desirable response to docetaxel [18, 19]. Overall, oncogenic PI3K/AKT signaling, whether intensified by PTEN loss or other pathway alterations, correlates with deficient hormonal agent responses and poor patient prognosis, while also predicting patient responsiveness to AKT inhibitors (capivasertib) (Table 1) [53].

### DNA damage response (DDR)

DNA damage response (DDR) pathways maintain genomic integrity and promote tumor suppression by correcting damage resulting from endogenous or exogenous DNA damaging agents [54]. While initial accumulation of DNA lesions initiates cell transformation and malign progression, DDR deficiency subsequently renders tumor cells more susceptible to DNA-damaging therapeutic agents [55]. DNA double-strand breaks (DSBs) are the most destructive lesions; thus, most DDR-targeted therapies focus on DSB repair mechanisms [56]. Homologous recombination deficiency (HRD) is attributed to incapacitation of a cell’s DSB-repair mechanisms using the homologous recombination repair (HRR) pathway [57]. It is estimated that HRR pathway mutations are present in 8 to 12% of localized PCa and 20 to 25% of CRPC [20]. An integrative study of somatic and germline variants of tissue specimens from 150 mCRPC patients reported that the most frequently altered HRR-related genes are BRCA2 (13.3%), ATM (7.3%), CDK12 (4.7%), and BRCA1 (0.7%). Notably, the distribution of these variants may vary between ethnicities [21, 58]. A recent cohort study from the phase III PROFound PARP inhibitor trial in mCRPC patients with HRD-related genes demonstrated that the patients with alterations in BRCA1, BRCA2, or ATM (at least one alteration) who received the poly (adenosine diphosphate–ribose) polymerase (PARP) inhibitors, olaparib, had better OS than those receiving enzalutamide or abiraterone and prednisone [22]. According multiple clinical trials, FDA has approved two PARP inhibitors, olaparib and rucaparib, as treatment for mCRPC in patients based on specific gene mutations [59]. Another retrospective cohort investigated the germline BRCA1/2 and ATM mutations in order to determine the progression status of PCa; as a result, patients with these mutations were shown to imply poor survival and younger age of death [60]. Development from localized PCa to mCRPC for BRCA2 germline mutation carriers with 5-year cancer-specific survival (CSS) is about 50–60% [61]. In addition, germline BRCA2 mutations in mPCa imply a worse prognosis than somatic mutations [62]. CDK12 has been implicated in transcriptional regulation of various HR-related genes, with CDK12 aberrations found in a subset of PCa (< 10%) [63, 64]. CDK12-altered PCa was characterized by a high Gleason score (> 8), worse survival, and an immunosuppressive tumor microenvironment [65]. The DNA mismatch repair (MMR) system also functions to preserve genome integrity by recognizing and repairing misincorporation errors, such as base–base mismatches, as well as small insertions/deletions generated during DNA replication and recombination [23]. Elimination of such errors prevents the accumulation of aberrations that could lead to DNA disruption. The MMR process incorporates complex interactions between MMR-specific proteins encoded by MSH2, MSH6, MLH1, and PMS2 and proteins of the DNA replication and/or recombination systems [66]. Mismatch repair deficiency (MMRd)-mediated PCa resulting from somatic mutations arises in nearly 5% of metastatic patients, with MSH2 and MSH6 mutations accounting for the majority of MMRd [24]. As reported by Rodrigues et al., MMR-defective *versus* MMR-proficient patients have shorter median OS: 3.8 vs. 7.0 years from initiation of luteinizing hormone-releasing hormone [25]. Deficiencies in MMR genes are associated with microsatellite instability (MSI) and increased tumor mutational burden causing increased tumor neoantigen expression and altered tumor specific T-cell responses [67]. Accordingly, CRPC patients with MMR deficiency reached objective responses and benefited from immune checkpoint blockade therapies [68]. In summary, DDR pathway gene alterations are important PCa biomarkers for established therapies and for clinical trials of agents inhibiting PARP or immune checkpoints (Table 1).

### Liquid biopsy

Liquid biopsy, the analysis of bodily fluids for biomarker detection, is a revolutionary approach opening new avenues for molecular profiling of PCa patients, including those with metastatic lesions. Liquid biopsies, most commonly derived via blood sampling, are minimally invasive, especially when compared to highly invasive trans-rectal and trans-perineal biopsies. Moreover, liquid biopsy can offer insights from early cancer diagnosis to relapse detection, prognosis, and therapy-response prediction [26]. Current strategies for liquid biopsy of various analyses include circulating tumor cells (CTCs), circulating tumor DNA (ctDNA), circulating tumor RNA (ctRNA), and extracellular vesicles (EVs) (exosomes), the latter of which contain important genomic, transcriptomic, and epigenomic information from PCa tumors (Table 1) [27].

#### CTCs and ctDNA

CTCs dislodge from primary or metastatic tumor tissue and have extravasated into the blood circulation, wherefrom they may act as precursors that initiate distant tumor metastases [69]. CTC enumeration (counting) constitutes a quantitative biomarker with predictive capacity regarding metastasis development, making it suitable for efficient disease monitoring. Molecular and genomic profiling of CTCs could mediate precise diagnosis and optimized therapy decisions for PCa patients. Moreover, CTCs may serve as prognostic biomarkers for mPCa patient survival and time-to-relapse [70, 71]. CTC enumeration correlates with PCa aggressiveness, with high CTC counts—commonly found in patients with bone metastasis—linked to poor prognosis and clinical outcome [65, 72]. Certain biomarkers are best detected via CTCs, including the ADT-resistance marker, AR-V7, which is most readily detected in the CTC component of liquid biopsies. Screening for AR-V7 mRNA derived from CTCs is more specific and sensitive compared to AR-V7 detection from cell-free circulating nucleic acids or from exosomes [73]. CTC analysis may in fact permit detection of the therapy resistance-associated AR-V7 variant at the protein level. Adding even greater molecular detail, subcellular protein localization data for AR-V7 in CTCs is proposed to enhance clinical utility, since nuclear AR-V7 in CTCs correlates most closely with CRPC status [74]. However, a recent study has shown that liquid biopsy-based AR-V7 detection correlates to poorer progression-free survival (PFS), with abiraterone and enzalutamide but better outcome in response to taxane-based chemotherapy [75].

While metastatic tissue biopsies are often relatively inaccessible in PCa, ctDNA derived from plasma or serum provides a non-invasive alternative for profiling of genomic alterations in mPCa [76]. Highly sensitive ctDNA-based screening of AR point mutations and gene amplifications has demonstrated strong associations between AR gene amplifications and CRPC, as previously found via invasive solid biopsies [77]. CtDNA-based screening for HRR changes has the utility to guide PARP inhibitor treatment of PCa [78]. Interestingly, discordances between DNA analyses derived from liquid *versus* tissue biopsy highlight the presence of more gene alterations—implying acquired resistance—in ctDNA than in tissue. This includes novel AR-activating alterations and subclonal BRCA1/2 secondary mutations and reversions [79]. Changes in tumor suppressors, such as PTEN and TP53, are also detectable via PCa ctDNA [80]. Undoubtably, CTCs and ctDNA are emerging as powerful biomarkers, with a current emphasis on patient screening and stratification for clinical trials. Currently, the commercially available CellSearch® system is the most widely used and only FDA-approved system for enriching and enumerating CTCs. It is based on epithelial cell adhesion molecule (EpCAM)-targeted CTC enrichment, which has limitations due to EpCAM downregulation during epithelial to mesenchymal transition (EMT), a process implied in CTC-genesis [70].

#### RNA-based fragments

RNA-based fragments including circulating ctRNA and messenger RNA (mRNA), as well as noncoding RNAs (ncRNAs) such as microRNA (miRNA) and long non-coding RNA (lncRNA), all carry transcriptomic information about cancer cells (Table 1). They are also easily accessible from plasma, serum, and urine [81]. The TMPRSS2-ERG fusion gene causes overexpression of ERG, accelerating progression of high-grade prostatic intraepithelial neoplasia (PIN) to invasive adenocarcinoma. Thus, TMPRSS2-ERG mRNA status in urine or blood samples is linked with some pathological features of PCa such as stage and Gleason score, and it can be introduced as a candidate marker for PCa development with progression and lower OS and PSA-PFS when treated with docetaxel [82, 83].

Numerous non-coding RNA (ncRNAs) are dysregulated in advanced PCa patients, enhancing the metastatic potential of tumor cell or driving it to acquire drug resistance. Notably, PCa antigen 3 (PCA3)—a lncRNA—is a urine-derived biomarker with clinical application in PCa, being overexpressed in 95% of men with primary and metastatic PCa tumors [84]. Combining PCA3 and TMPRSS2-ERG gene fusion tests has been suggested as a means to optimize PCa detection, reducing the necessity of invasive biopsies [85]. More specifically, upregulated lncRNA-MALAT1 has also been linked to CRPC progression [86]. According to an investigation by Wang et al., lncRNA-MALAT1, by promoting the production of AR-v7, contributes to enzalutamide resistance [87]. Another study suggested that MALAT1 may also have a role in docetaxel resistance through interaction with AKAP12 indirectly [88]. Multiple miRNAs have been explored in serum and plasma of patients with mCRPC. In particular, increased levels of miRNA-141-3p have been linked with progression and metastasis of PCa and shorter OS in mCRPC patients [89–92]. It has also been suggested that higher levels of miR-375 constitutes prognostic and predictive biomarkers, implying poor OS, guiding CRPC staging, and showing resistance in docetaxel [90, 92–94]. However, evidence on the diagnostic and prognostic value of miRNA is conflicting, and at the present time, no clinically miRNA-based test is available for PCa.

#### Exosomes

Exosomes of endosomal origin are enriched with various protein, RNA and DNA fragments, as well as bioactive lipids; all of which are detectable in biological fluids. Exosome cargo is representative of tissue-of-origin, thus serving as a potential biomarker for diagnosis and monitoring of PCa progression and treatment-response [95, 96]. However, as with other liquid biopsy-derived analytes, challenges impacting clinical applications center on the (lack of) optimization and standardization for detection approaches. These challenges are acute in exosome analysis, where detection, purification, and isolation are difficult due to small particle sizes and low densities [95]. Nevertheless, exosome analysis in PCa is developing, with detection of lncRNAs and other PCa biomarkers recently reported, including analysis of prostate-specific membrane antigen (PSMA) from urine-derived exosomes (Table 1) [27].

## Phenotypic biomarkers

Due to the heterogeneity and complexity of advanced PCa, genotypic biomarker use for precision medicine remains challenging. On the other hand, phenotypic biomarkers, based on observable characteristics, may simplify decision-making and boost precision medicine in advanced PCa. Integrating phenotypic and genotypic biomarkers could further add to an in-depth understanding of PCa pathology, as well as to the development of potent targeted therapies.

### Protein biomarkers

Measuring the changes of some proteins can be a valuable tool and is easily accessible in cancer prediction and diagnosis. Some of the common protein biomarkers are detailed below:

#### Serum testosterone levels

A retrospective analysis of CRPC patients evaluated the impact of serum testosterone levels to guide a decision on optimal treatment for patients who received androgen receptor-targeted agents (ARTAs). In the subgroup of patients with testosterone levels between 5 and 50 ng/dL treated with abiraterone or enzalutamide, PSA-PFS and OS were longer than those with levels below 5 ng/dL group. Nevertheless, patients with a testosterone level below 5 had a prolonged PSA-PFS and higher PSA response with abiraterone than enzalutamide [28]. A meta-analysis study also showed that patients with CRPC with higher testosterone levels were associated with significantly greater OS and PFS treated with ARTAs than patients with lower testosterone levels [97]. In another study, a subgroup of patients with CRPC receiving enzalutamide with testosterone levels > 0.05 ng/mL compared to those levels < 0.05 ng/mL showed a significant superiority for PFS and a trend of superiority for OS but not significant, while for patients treated with docetaxel, PFS was worse. For abiraterone, no difference was noted in PFS or OS in different level of testosterone. Thus, pre-treatment serum testosterone levels may have predictive value in the selection of AR-targeted therapy versus chemotherapy in patients with mCRPC [29].

#### SLFN11 expression

The Schlafen family member-11 (SLFN11), a DNA/RNA helicase, which is commonly detected in CTCs is linked with the activity of DDRs [98]. Conteduca et al. assessed the impact of SLFN11 expression in mCRPC patients treated with platinum-based chemotherapy. Patients with overexpression of SLFN11, which included approximately 45% of metastatic CRPC patients and 25% primary PCa, treated with platinum chemotherapy showed a longer radiographic PFS and a decline of PSA by 50% or more, while no difference in OS was seen [30]. Moreover, Scher and colleagues showed that CTC expression of SLFN11 in patients with advanced PCa supported the predictive role of SLFN11 expression in selecting patients who may benefit from platinum chemotherapy and PARPi [98].

#### Total alkaline phosphatase (tALP)

Sonpavde and colleagues evaluated the association of serum alkaline phosphatase (ALP) changes with OS in CRPC patients with bone metastases and elevated ALP (≥ 120 u/L) treated with docetaxel or mitoxantrone. Patients whose ALP normalized to < 120 IU/L at 90 days after treatment had a better median OS compared to those without normalization (18.8 vs. 13.4 months). Nevertheless, increasing ALP was correlated with notably poorer survival compared to those without an ALP increase (10.5 months vs. 15.3 months) [31]. Furthermore, a normal pre-treatment total ALP and a reduction of 10% or greater in an elevated baseline tALP after 4 weeks of treatment with Radium-223 are linked to longer OS [99]. In a study conducted by James et al., it was found that patients with ALP decline after 12 weeks of treatment with Ra-223 showed improved OS. Therefore, changes in tALP levels may be a prognostic marker for estimating OS [100].

### Neutrophil-to-lymphocyte ratio (NLR)

A simple-to-calculate marker, the neutrophil-to-lymphocyte ratio, has been investigated as a prognostic tool for patients with CRPC in multiple studies. According to Murata et al., patients with higher pre-treatment NLR (cut-off = 3.76) who were receiving abiraterone had a worse OS [32] Similarly, Kumano et al. [33] reported that patients with CRPC receiving enzalutamide had inferior OS and CSS when their pre-treatment NLR was higher (cut-off of 2.14) compared to those with a lower NLR. In contrast, Loubersac et al. [101] suggested that baseline NLR changes during therapy are not a sufficient predictor in response to abiraterone and should not be used to guide treatment [101]. Likewise, a recent study investigated the importance of baseline NLR and its alterations during the treatment of metastatic PCa patients with ^223^Ra or docetaxel. Patients with low NLR ≤ 5 at baseline treated with ^223^Ra, but not docetaxel, had a better median OS (14.5 months) compared with the high NLR > 5 (8.5 months). After 12 weeks of therapy with ^223^Ra, patients with NLR ≤ 5 at baseline had a longer median OS compared with the NLR > 5 group (15 vs. 9.5 months). Patients with NLR ≤ 5, whose NLR at baseline remained constantly low, had a significantly longer median OS than patients whose NLR increased to NLR > 5 (16.0 vs. 9.1 months) [102]. Another study [103] assessed patients treated with docetaxel before or after AR-directed therapy. Patients with NLR ≥ 2.5 versus those with NLR < 2.5 had lower 1-year radiographic PFS (rPFS) and 2-year CSS. Patients with NLR < 2.5 showed better 1-year rPFS and 2-year CSS rates who received post-docetaxel ARAT agents compared to patients with pre-docetaxel ARAT. In patients with an NLR of 2.5 or higher, the order of docetaxel and AR inhibitors did not affect rPFS or CSS. The NLR assessment in CRPC patients may consider a putative marker for guiding the sequence of therapy with docetaxel and ARAT [103].

### Tumor pathology as a gold standard predictor of mPCa

Immunophenotyping methods focusing on the tumor microenvironment (TME) have allowed prediction of tumor progression and dynamic therapy response in PCa patients. Multiplexed immunohistochemical (IHC) staining techniques, as well as multiplexed immunofluorescence (IF), combined with powerful analysis software tools, which provide automated cell segmentation and histological classification by Machine/Deep Learning, now enable assessment of numerous protein biomarkers simultaneously and in spatial context, generating more comprehensive prognostic and predictive information with potential to transform personalized clinical management (Table 1) [34]. The current status quo of digital pathology refers to digitizing of slides with subsequent visual analysis – done by looking at monitors rather than through a microscope’s oculars. With the advent of computational pathology, also referred to as next-generation digital pathology [34], digital analysis is about to become a game changer in pathology as well as pharmacological research, as such systems allow to perform digital analysis and in particular high-plex analyses for determination of molecular correlations using machine learning and especially deep learning algorithms [104]. For example, in metastatic PCa, PTEN genomic loss is predominantly identified by fluorescence in situ hybridization (FISH). Yet, PTEN inactivation may result from alternate causes including dysregulation of gene expression or function-altering mutations. IHC of PTEN in formalin-fixed paraffin-embedded tissues (FFPE) can provide additional and more direct information on protein levels and localization, supporting selection of targeted therapies [105]. Similarly, AR expression assessment via digital pathology may add expression and localization information to guide the use of therapies like ARSIs, or when evaluating alternative treatments after the emergence of ADT-resistance. As such, computational pathology may differentiate AR-null from AR-expressing PCa in a metastatic context [106].

Programmed death-ligand 1 (PD-L1), which is predominantly expressed on the surface of tumor cells, promotes immune-evasion and negative regulation of T-cells, through binding to the programmed death 1 (PD-1) receptor on T-cells. Therapies blockading this immune checkpoint have induced clinical responses in PCa. Yet clearly some patients benefit significantly from anti-PD-1/PD-L1 therapeutics, making imperative the identification of a predictive biomarker for this treatment. To this end, some evidence suggests that PD-L1 expression level, hypermutated or microsatellite-unstable status, and/or DNA-repair deficiencies predict poor checkpoint inhibitor responses [68]. In fact, several studies have investigated the prognostic value of PD-L1 expression in tumor cells. PD-L1-positive tumor/FFPE correlated with higher risk of progression in metastatic patients with lymph-node-positive PCa, as well as higher risk of recurrence after radical prostatectomy [35, 107]. Of note, the dMMR status can also be determined phenotypically by the loss of MMR protein in advanced PCa. In this context, recent studies report that 5% to 12% of mCRPC patients may benefit from immune checkpoint–inhibiting therapies. Thus, IHC staining to detect loss of MMR protein could constitute a predictive biomarker for immune checkpoint-inhibiting immunotherapeutics [25]. Despite current controversies and challenges in modulating the immune system as a therapeutic approach, promising treatment directions are emerging. Appropriate application of these ‘now on demand’ improved biomarkers supported by data from well-powered clinical analyses can serve as complementary biomarkers for these emerging new therapies.

### Improved imaging modalities for early PCa detection

Improvements in imaging techniques and accessibility to imaging equipment have had a significant impact on staging, treatment, and assessment of therapeutic response in PCa patients in recent years. Multiparametric magnetic resonance imaging (mpMRI) is now routinely used for guiding biopsies and differentiating significant vs indolent disease [108, 109]. MRI is being increasingly used for defining anatomy for radiotherapy treatment planning, and quantitative imaging biomarkers have also shown the potential to spatially define tumor heterogeneity [110–112]. The introduction of advanced imaging techniques, such as radiolabeled prostate-specific membrane antigen (PSMA) and choline combined with positron emission tomography (PET), computed tomography (CT) or MRI, has significantly enhanced the personalized management of metastatic PCa. These imaging modalities provide improved performance and accuracy in detecting and monitoring metastases, allowing for more effective treatment strategies. PSMA—a trans-membrane protein—is a diagnostic phenotypic biomarker highly expressed in the metastatic and hormone-refractory status of PCa cells [113]. Radioactive tracers-based PSMA has been confirmed as a potential target for PET/CT imaging. PSMA-PET/CT imaging has high performance to detect metastatic patients, bone lesions, and a higher lymph node detection rate [113]. Interestingly, choline, a component of phosphatidylcholine, is considered a potential prognostic biomarker resulting from the high concentration of it in PCa cells [114]. PET/CT imaging, utilizing radio-labeled choline, has demonstrated remarkable sensitivity (94-100%) and specificity (66.7-99.7%) for lymph node staging. Thereby, it can potentially address some limitations associated with conventional imaging techniques in the diagnosis and staging of PCa [115]. Moreover, choline-PET/CT significantly improved the detection of bone metastases with more than 90–95% sensitivity and 92 to 99% specificity, although there is a controversy about to use of MRI and choline-PET/CT in detecting bone metastasis. A meta-analysis study of advanced PCa patients has compared the MRI to choline-PET/CT and BS for detecting bone metastases; the result showed MRI with 97% sensitivity, and 95% specificity has a better performance [116].

With the addition of artificial intelligence (AI) approaches, a spatial map of tumor heterogeneity enables a precision-based approach to radiotherapy, coined biologically targeted radiation therapy [117••, 118]. Another modality which has shown value in all the stages of the RT workflow is ultrasound. Besides the possibility to segment and track (in real-time) prostate tumors, especially with the advent of AI and robotics to improve the efficiency and the reliability of the process [119], Doppler and contrast ultrasound imaging can be used to image tumors’ vasculature. Metrics of the vasculature are becoming established as useful imaging biomarkers of cancer and prognosis, improving sensitivity and specificity of cancer detection. New microbubble ultrasound contrast agents targeting molecules overexpressed by tumors pave the way to using ultrasound as a molecular imaging technique [120]. Moreover, ultrasound elastography and ultrasound tissue characterization have also elicited growing interest in RT as they can provide either a measure of stiffness of the tumor or characterization of tumor tissue micro- and macrostructure [121]. While treatment response is routinely monitored using periodic measurement of serum PSA, the long interval between the first rise in PSA reading and diagnosis of localized recurrent disease can be prolonged, increasing the risk of development of metastatic disease. Quantitative MRI, with or without PSMA PET, has shown promise for early detection of localized disease and the opportunity for effective salvage therapies [117••, 122].

Other emerging optical imaging technologies including using photonic devices and Raman Spectroscopy have also been developed to detect the presence of PCa cells [123]. A portable rectal near infrared (NIR) scanner utilizing photonic sensing combined with optical tomography was developed which offers the potential of a minimally invasive, high-spatial resolution screening tool for PCa. The other significant advantage of the rectal NIR scanner is that it offers a comparatively low-cost and simple setup when compared to MRI. Unlabeled, unfrozen, human prostate tissues including both normal and cancerous prostate tissue (the latter being surrounded by normal tissue) was examined. The study found that adenocarcinoma, or glandular cancer, could be imaged in 3D without the use of contrast agents up to a depth of 3 mm [124].

An optical photonic-crystal based biosensor was also recently demonstrated, which was able to distinguish in vitro various PCa and noncancerous cell lines (i.e., RWPE-1, BPH-1, PC-3, DU-145, and LNCaP). This study was able to quantify the refractive index (RI) properties of the different cell lines and reported that there is a correlation between cancerous cells having a smaller RI versus noncancerous cells [125].

Finally, another optical technique, Raman spectroscopy, has been successfully employed recently in order to identify PCa patients suffering radiation toxicity following radiotherapy. Blood samples were collected as part of a trial conducted in Ireland from patients enrolled in a prospective, phase II non-randomized controlled clinical study. Peripheral blood mononuclear cells (PBMC) were isolated from 42 patients who had undergone radiotherapy to treat PCa and exhibited either severe or no/minimal late radiation toxicity. Raman spectroscopy of lymphocytes was performed, and radiation response was assessed by examining a total of 50 spectra collected from each of the unirradiated and irradiated samples from each patient. Using the known radiation toxicity scores, a discriminant analysis model was developed by the authors of this study. Based on this analysis, Raman spectroscopy was able to achieve a sensitivity of 95% and specificity of 92% demonstrating the potential for the method to be used as a means of individualized patient radiotherapy. By providing non-invasive or minimally invasive measures, medical imaging constitutes a crucial, phenotypically oriented pillar for patient monitoring and therapy-response assessment over disease course [126].

## Conclusions

Advanced PCa is currently not a curable disease. Nevertheless, precision medicine promises effective ways to guide improved decision-making; linking an ever-increasing array of targeted therapies to the patients that will benefit from them most. Elaboration of biomarkers not only enables the optimized application of existing therapies, but also the improved stratification of patients into trials of new therapies, increasing the potential to detect efficacy with therapies whose mechanisms match only specific patient subsets. Overall, analyzing altered molecular pathways illuminates biomarkers that can map disease progression and guide personalized, adaptable therapy application. In partnership with these molecular approaches, medical imaging techniques and AI-empowered image cytometry software are increasing in their capacity to sensitively monitor patient responses. Thus, molecular and phenotypic biomarker monitoring are emerging as complementary counterpoints in the dynamic, longitudinal management of PCa patients over disease course. We believe that this interplay, forming the guidance mechanism for precision medicine in PCa, will transform the prospects for patients with this disease. Nevertheless, so far only a few of the discussed potential biomarkers are part of routine diagnostics in PCa. There is more work needed to identify the most informative biomarkers together with the development of standardized, practical diagnostic tests and analytical software to screen for them on a larger scale and to progress their use into routine patient care.
